# Sentience and Intrinsic Worth as a Pluralist Foundation for Fundamental Animal Rights

**DOI:** 10.1093/ojls/gqad003

**Published:** 2023-02-18

**Authors:** Jane Kotzmann

**Affiliations:** Alfred Deakin Postdoctoral Research Fellow in Law, Deakin University Law School, Melbourne, Victoria, Australia

**Keywords:** Sentience, intrinsic worth, intrinsic value, dignity, animal rights, foundation

## Abstract

To date, welfare protections have failed animals. In this context, many animal advocates and scholars have supported recognition of animal rights. Animal rights theory, however, remains underdeveloped. This article contributes to the development of animal rights theory and, in this respect, proposes the utilisation of sentience and intrinsic worth concepts as a pluralist foundation for prospective animal rights. Sentience and intrinsic worth as a conceptual underpinning for animal rights hold clear benefits in that (i) the concepts are already embedded in many legal systems, (ii) sentience would enable the development of animal rights to be built on the established interest theory of rights, and (iii) sentience directly links to the justification of rights as being primarily concerned with the prevention of pain and suffering.

## 1. Introduction

[T]he question of grounds remains to be faced: what are the conditions that make it the case that anything is a right of this kind, and are they ever satisfied?^1^

Recent decades have seen an increased interest in, and awareness of, the lives of animals. While animal welfare laws were first introduced in the UK in 1822,^2^ modern animal ethics dates to the 1970s.^3^ Peter Singer’s book *Animal Liberation* (1975) inspired people to seriously consider whether the human treatment of animals in various contexts is humane.^4^ Given that many animals have their own subjective experiences of the world and can have positive and negative feelings just like humans,^5^ reflection on this question coupled with an awareness of animal law leads to the almost inescapable conclusion that many animals experience unnecessary pain and suffering because of lawful human treatment.^6^ Many animal law scholars have argued that animals need rights to adequately protect them from human harms.^7^ Although there is no broad consensus that animals need rights, it does seem clear that contemporary animal welfare laws have proven insufficient for the purpose of protecting animals. This is evident from the failures of welfare legislation to ‘protect most animals from routine and systematic ill treatment’^8^ and the consistent prioritisation of human interests over animal interests.^9^

In this context, numerous legal systems have introduced and changed laws to better protect animals. Changes have included stronger animal welfare laws, including increased criminalisation and penalties. For example, in 2008 the South Australian Parliament amended the Animal Welfare Act 1985 (SA) to increase the maximum penalties for animal welfare offences, which doubled the fines and prison sentences subsequently handed down by courts.^10^ Some countries have amended their constitutions to improve the situation of animals. In 2002, for instance, Germany amended its Basic Law to include a provision that obliges the state to protect animals.^11^ Further, several civil law jurisdictions have enacted provisions asserting that animals are not the same as other ‘things’.^12^ One notable way that countries have amended their laws is to legally recognise animal sentience. The European Union first recognised animals as sentient in 1997 in a protocol to the Treaty of Amsterdam.^13^ Since then, diverse jurisdictions, including Tanzania,^14^ New Zealand,^15^ Quebec,^16^ Oregon (United States),^17^ the Australian Capital Territory (ACT)^18^ and Spain,^19^ have enacted laws recognising that animals are sentient. Another significant way that jurisdictions have altered their laws is to recognise the concept of animal intrinsic worth. Various terms have been used in this respect, including ‘dignity of animals’ and ‘intrinsic value’.^20^ These terms are suggestive of a general idea that animals have worth in and of themselves, outside of their utility to humans.^21^

A small number of countries have gone further and recognised some animal rights. To date, courts in Argentina,^22^ Colombia,^23^ India^24^ and Pakistan^25^ have established some animal rights. These rights include the right to habeas corpus, which protects against unlawful imprisonment, the right to life and the right to freedom from torture.^26^ These developments, together with scholarship in animal law, suggest that ‘Legal animal rights are on the horizon’.^27^ While animal rights appear to be on the way, little work has been done to develop the theory to underpin animal rights. As Saskia Stucki asserts, ‘there is a need for … a theory of animal rights as *legal* rights [and] … the nature and conceptual foundations of legal animal rights remain remarkably underexplored’.^28^ Theory in this respect is important because it works to both explain and guide the development of the law.^29^ Moreover, theory would also operate to underpin the legitimacy of legal animal rights in the first place.

This article is concerned with ‘fundamental animal rights’, a conceptual category introduced by Stucki.^30^ As Stucki explains, fundamental animal rights are legal rights similar in nature and purpose to human rights and are characterised by ‘substantive fundamentality and normative robustness due to their reduced infringeability’.^31^ Fundamental animal rights might include, for example, a right not to be subject to torture or a right to liberty. While it could be argued that animals hold some legal rights,^32^ fundamental animal rights in the sense outlined by Stucki do not yet exist.^33^

This article seeks to contribute to the development of animal rights theory and, in pursuing this purpose, focuses on the theoretical foundation of prospective animal rights. In this respect, it argues for utilisation of the concepts of sentience and intrinsic worth^34^ as a pluralist foundation for the development of legal animal rights. Employing these concepts to underpin animal rights has three clear advantages. First, the concepts of animal sentience and animal intrinsic worth are already recognised in many legal systems. Second, sentience aligns with the interest theory of rights because a sentient being has interests.^35^ Third, a sentient being has the capacity to experience negative states like pain and suffering. This article contends that fundamental rights are primarily concerned with the prevention of individual pain and suffering.^36^ Locating sentience at the foundation of animal rights aligns with this conception of the core purpose of rights. Moreover, recognising animal intrinsic worth in conjunction with sentience is necessary to establish that animal pain and suffering matters.

The concept of legal animal rights raises many other theoretical questions. For example, it might be asked ‘whether animals are the kind of beings who can potentially hold legal rights’,^37^ what rights animals should have and whether all animals are entitled to the same rights. While interesting, these questions lie outside the scope of this analysis. This article proceeds as follows. Section 2 examines the way concepts of animal sentience and intrinsic worth are already embedded in the law in many jurisdictions. Section 3 explores the connection between sentience and the interest theory of rights, which is used as the basis for the account of animal rights set out in this article. Section 4 discusses the connection between sentience and prevention of pain and suffering. In section 5, some further reflections on the utilisation of sentience and intrinsic worth as a pluralist foundation for animal rights are discussed.

## 2. Legal Recognition of Animal Sentience and Intrinsic Worth

### A. Meaning of Sentience

Broadly, sentience refers to a capacity to subjectively experience the world, including experiencing positive emotions or states such as happiness and joy, and negative states like pain and suffering.^38^ Scientific methods are used to determine whether a particular species is sentient. These methods look for characteristics such as the possession of nociceptors, the capacity of the brain to receive information from different sensory sources, neural pathways linking the nociceptors to integrative parts of the brain, behavioural responses to various stimuli, self-protective behaviour, formation of associations and attaching value to pain relief.^39^ Rather than being sentient or not sentient, humans and animals are sentient to varying degrees; some animals have greater capacity for awareness and emotions than others.^40^ It is generally agreed that all vertebrates are sentient,^41^ and studies in relation to ‘amphibians, reptiles, fish, cephalopods and decapod crustaceans’ indicate that they are also sentient.^42^ Nevertheless, some ambiguity regarding the scope and detail of the meaning of sentience remain.^43^ For the purposes of this article, David J Mellor’s definition of sentience as ‘a capacity to consciously perceive negative or positive sensations, feelings, emotions or other subjective experiences which matter to the animal’ is adopted.^44^

### B. Meaning of Intrinsic Worth

The term ‘intrinsic worth’ broadly refers to the concept that something or someone has value in and of themselves.^45^ In relation to animals, it is used to indicate a view that animals themselves have value, outside of any value they might hold for humans.^46^ The concept of intrinsic worth is broadly synonymous with ‘intrinsic value’, ‘inherent worth’ and ‘inherent value’. It also speaks to the general meaning of dignity, or inherent dignity. Although, as Will Kymlicka identifies, ‘dignity is not the natural language of [animal rights] … theory’,^47^ dignity can be conceived to apply to all individuals that are able to grow and develop, including animals.^48^

### C. Animal Welfare Legislation Is Minimally Underpinned by Implied Recognition of Animal Sentience and Intrinsic Worth

Globally, most jurisdictions have animal welfare (or protection) laws in force. Animal welfare laws date back to early legislation, including the Act against Plowing by the Tayle, and Pulling the Wooll off Living Sheep enacted by the Parliament of Ireland in 1635,^49^ the Puritans of Massachusetts Bay Colony Code of 1641,^50^ and the Cruel Treatment of Cattle Act of 1822 and the Cruelty to Animals Act of 1849 passed by the British Parliament.^51^ While a small number of early statutes sought to prevent cruelty to animals, anti-cruelty legislation did not become commonplace until the 19th century.^52^ In contemporary times, animal welfare laws seek to prevent or minimise harm to animals by criminalising human conduct towards animals and, in some cases, imposing obligations on humans for the care of animals.^53^

Although most contemporary animal welfare legislation does not include the word ‘sentient’, it is arguable that much of it is underpinned by an implicit recognition of animal sentience.^54^ This recognition may represent a narrow understanding of sentience as equivalent to suffering, or a broader one amounting to capacity to experience negative and positive states. In this respect, Lora Dunn and David B Rosengard assert that ‘Without being sentient, animals cannot suffer; sentience is therefore implicitly at the root of … animal protection laws’.^55^ By way of example, the Congressional Statement of Policy within the United States’ Animal Welfare Act of 1966 indicates that animals in particular settings must be ‘provided [with] *humane* care and treatment’.^56^ If animals were incapable of experiencing negative emotions like pain and suffering, it may be irrelevant whether human treatment of them was humane or otherwise.

Reference to animal emotions or their capacity for emotions in animal welfare legislation may signify an implicit acknowledgement of animal sentience. For example, § 1 of the Finnish Animal Welfare Act 1996 states that the Act aims ‘to protect animals in the best possible way from suffering, pain and distress’.^57^ Acknowledging that animals *can* experience suffering, pain and distress constitutes an acceptance that animals are to some degree sentient. Similarly, by indicating that it protects animals that ‘have developed senses to receive external stimuli and a developed nervous system to feel painful external influences’,^58^ the Animal Protection Act 1999 of Slovenia accepts that such animals are at least minimally sentient. Comparable examples of legislation that appear to implicitly recognise animal sentience in other jurisdictions abound.^59^

Similarly, contemporary animal welfare legislation may be interpreted to rest on a view that animals have intrinsic value.^60^ This may be the case where legislation, or the relevant legislative history, indicates that the protections it provides are for the benefit of the animal rather than the human owner or the public more generally. This interpretation is supported by the judgment in *State v Crow*,^61^ in which the Court of Appeals of Oregon held that the relevant legislation evinced an intention to protect individual animals instead of the public.^62^ On this basis, the Court held that the trial court did not make a mistake in entering 13 separate convictions for unlawful possession of an animal.^63^ Similarly, in *National Society for the Prevention of Cruelty to Animals v Minister of Justice and Constitutional Development*,^64^ the Constitutional Court of South Africa stated that ‘the rationale behind protecting animal welfare has shifted from merely safeguarding the moral status of humans to placing intrinsic value on animals as individuals’.^65^

### D. Legal Developments towards the Express Recognition of Animal Sentience and Animal Intrinsic Worth

While animal welfare legislation may be underpinned by an implied recognition of animal sentience, recent decades have seen the emergence of a trend amongst mostly Western legal jurisdictions towards expressly recognising animal sentience in the law.^66^ The European Union was the first to recognise animal sentience in a protocol to the Treaty of Amsterdam.^67^ Subsequently, recognition of animals as ‘sentient beings’ was included in the Treaty on the Functioning of the European Union in 2008.^68^ In the same year, Tanzania recognised animals as sentient beings in its Animal Welfare Act 2008.^69^

Numerous jurisdictions have followed this lead and passed their own animal sentience recognition provisions. In North America in 2013, Oregon passed legislation which states that ‘animals are sentient beings capable of experiencing pain, stress and fear’.^70^ Similarly, in 2015, Quebec enacted laws stating that ‘animals are not things. They are sentient beings and have biological needs.’^71^ In South America, Chile (2009), Peru (2016) and Colombia (2016) have all enacted laws recognising animal sentience. For example, Colombia’s Law 1774 provides that ‘Animals as sentient beings are not things, they will receive special protection against suffering and pain, in particular, suffering and pain caused directly or indirectly by humans’.^72^ In Europe, the Netherlands (2011),^73^ Lithuania (2012),^74^ France (2014),^75^ Sweden (2018),^76^ Brussels (in Belgium, 2018),^77^ Denmark (2021)^78^ and Spain (2021)^79^ have all recognised animal sentience in their laws. In Oceania, New Zealand recognised animal sentience in 2015,^80^ while the ACT did so in 2019.^81^ Recently, the UK passed the Animal Welfare Sentience Act 2022 (UK), which not only recognises animals as sentient beings, but also establishes an Animal Sentience Committee, responsible for scrutinising government policy to determine whether it has taken adequate account of animal welfare.^82^

Similarly, the intrinsic value of animals, or animal dignity, has been recognised in law. In 1992, Switzerland amended its constitution to include the concept of ‘dignity of the creature’, initiating a debate on the potential applicability of the dignity concept to animals.^83^ Subsequently, in 2008, it amended its Animal Protection Act to recognise animal dignity. The purpose of the Animal Protection Act is expressly stated as the protection of ‘the dignity and welfare of animals’,^84^ with dignity being defined as ‘the inherent worth of the animal that must be respected when dealing with it’.^85^

Several other jurisdictions have also legally recognised the intrinsic worth of animals. For example, the autonomous region of Brussels included the phrase ‘dignity of the animal’ in its Animal Protection Act of 2018.^86^ Similarly, the Netherlands has recognised animal dignity in the Animals Act 2011, which provides that ‘The intrinsic value of the animal is recognized’.^87^ In similar terms, Norway recognises the ‘intrinsic value’ of animals in its Animal Welfare Act of 2009.^88^ In Australia, section 4A(1) of the Animal Welfare Act 1992 provides that ‘animals have intrinsic value and deserve to be treated with compassion and have a quality of life that reflects their intrinsic value’.^89^ Comparable references can be found in legal jurisprudence. For example, in *Balakrishnan v Union of India*, the High Court of Kerala stated that ‘Though not homosapiens, [animals] are also beings entitled to dignified existence and humane treatment sans cruelty and torture’.^90^ Similarly, in the case brought by the Nonhuman Rights Project on behalf of Happy the elephant, Justice Tuitt of the Supreme Court of New York asserted that Happy was ‘an intelligent autonomous being who should be treated with respect and dignity’.^91^

### E. Developing Legal Definitions of Sentience and Intrinsic Worth

The foregoing discussion has established that the concepts of animal sentience and intrinsic worth are embedded in many legal systems, sometimes implicitly and sometimes explicitly. Different jurisdictions have, however, taken different legislative approaches to the recognition of these concepts. Moreover, the definitions of these concepts are likely to evolve over time.

While some legal provisions recognising animal sentience include a definition within the legislation, some legislative definitions do not canvass the full contemporary meaning of sentience. For example, although the legislation enacted in Oregon acknowledges that ‘animals are sentient beings capable of experiencing pain, stress and fear’,^92^ it overlooks the capacity of sentient animals to experience positive states.^93^ Further, some legislation acknowledges sentience without defining what it means at all.^94^

Although sentience is fundamentally a scientific term, its legal meaning will ultimately be determined by the legislature, the courts or both. When drafting legislation, legislators have a choice as to whether to exhaustively define a term; non-exhaustively define a term, thus leaving it open to further interpretation; or leave the term undefined and subject to courts’ interpretation. When interpreting legislation, courts ordinarily look at the plain meaning, the specialised meaning and the purpose of the words.^95^ To the extent that the term ‘sentience’ is understood as a specialist term of science, it might import related scientific concepts.^96^ This might extend to relying on science to determine which animals are sentient and thus should be protected by the law—and by extension which animals will not be protected. Some scholars have expressed concern that this will place too much emphasis on science, which itself may be subject to bias and remains incomplete.^97^ Ultimately, however, to the extent that legislatures leave the term ‘sentience’ undefined, it is for courts to interpret the meaning of sentience considering its legislative purpose.^98^ Over time, judicial consideration should result in the development of a legal understanding of the term.

Legal recognition of animal sentience alone does not necessarily imply that animals possess any intrinsic or inherent value. Although sentience may be considered to give rise to intrinsic value, they are two separate concepts. Despite this, a legislative text together with its drafting history may lead a court to conclude that the legal recognition of animal sentience represents an acknowledgement or implies that animals have their own intrinsic value. This might be the case, for example, if the drafting history indicated an intention to recognise animal sentience because this is the basis for animals’ intrinsic value.^99^ Further, as noted above, some provisions recognising animal sentience also expressly state that animals have intrinsic value. The two concepts of sentience and intrinsic worth are thus separate and yet closely connected.

Where legislation does expressly recognise animals’ intrinsic worth, it might do so in various ways. As noted above, the terms ‘intrinsic worth’, ‘intrinsic value’, ‘inherent worth’, ‘inherent value’ and ‘dignity’ are generally synonyms in this respect. Even so, these remain somewhat vague concepts. Their lack of clarity is highlighted by criticism directed at the term ‘dignity’ in the context of human rights. In this context, dignity has been attacked on the basis that it is ‘vacuous’ and without any real meaning.^100^

Nevertheless, some legislatures have defined the concept of intrinsic worth in relation to animals in a way that gives the phrase concrete meaning. As discussed, the Swiss Animal Protection Act defines dignity as meaning ‘the inherent worth of the animal that must be respected when dealing with it’. It then goes on to explain that where ‘any strain imposed on the animal cannot be justified by overriding interests, this constitutes a disregard for the animal’s dignity’. Further,

Strain is deemed to be present in particular if pain, suffering or harm is inflicted on the animal, if it is exposed to anxiety or humiliation, if there is major interference with its appearance or its abilities or if it is excessively instrumentalised.^101^

Dignity has also been operationalised by Swiss Courts, with the Swiss Federal Court holding that certain experiments on primates were not consistent with respect for their dignity.^102^ Similarly, recognition of animals’ intrinsic value in the Netherlands’ Animal Protection Act is defined as meaning ‘recognition of the self-esteem of animals, being sentient beings’.^103^ The explanatory memorandum to the Act further elucidates the concept as ‘representing the uniqueness of the animal as a living creature’.^104^

While the concept of animals’ intrinsic worth is somewhat imprecise, the way it has been used in law seems to suggest a shift in the human perception of animals. Pursuant to animal welfare paradigms, animals are generally seen as having predominantly instrumental value. Recognition of animals’ intrinsic worth or dignity, in contrast, ‘seems to be construed as an aspect separate from [welfare] … having little to do with the experience of pleasure and pain’.^105^ In other words, recognition of animals’ intrinsic worth is indicative of a growing view that animals have value in and of themselves, outside of their value to humans.

### F. The Utility of Building on Known Concepts

This article asserts that sentience and intrinsic worth provide appropriate concepts on which to base legal animal rights. One reason for this argument is that these concepts are already embedded in the law. This provides an advantage in that it can take significant amounts of time to achieve legal change. Indeed, campaigns for the legal recognition of animal sentience first originated in 1988, when Compassion in World Farming advocated for the recognition of animals as ‘sentient beings’ in the Treaty of Rome of the European Union.^106^ This first campaign was ultimately successful in that the Treaty of Amsterdam of 1997 included a Protocol recognising animals as ‘sentient beings’.^107^ However, nine years is a considerable time to wait for change. The embeddedness of sentience and intrinsic worth recognition within the law also provides an advantage in that it reflects general agreement by the public that animals are sentient and have intrinsic value, and can be used as evidence of this agreement. In other words, the existing logic that animals are sentient and thus deserving of legal protection is already found in the law and provides a ready springboard for the construction of animal rights.

On the other hand, the argument that sentience could provide a foundation for animal rights may be challenged on the basis that early proponents for the moral relevance of animal sentience, including Jeremy Bentham, did not believe in rights. For example, Bentham famously queried why ‘the law [should] refuse its protection to any sensitive being’,^108^ but nevertheless labelled rights as ‘nonsense upon stilts’.^109^ Bentham’s concern was that recognising natural rights that were not set out in the law would lead to social unrest. Instead, he argued that legislators should seek to directly use the law to maximise happiness. Bentham’s approach, however, fails to explain how legislators would go about assessing what laws would maximise happiness. This is where a concept of rights that is based on both interests derived from sentience as well as intrinsic worth has utility. It would provide legislators with a ready reference guide to the kinds of laws that would serve animal interests. At the same time, it would also provide a set of moral standards against which to evaluate governments, which may not always act in the best interests of animals.

## 3. Sentience and the Interest Theory of Rights

The second reason that sentience is a suitable foundation for animal rights is that it aligns very usefully with the interest theory of rights. This section will explore the connection between sentience and the interest theory of rights, as well as the extension of the interest theory to animals in the literature. Based on this discussion, this article will assert that sentience can act as a *de facto* standard to determine rights eligibility under the interest theory of rights.

### A. The Connection between Sentience and the Interest Theory of Rights

The emergence of sentience recognition as an important aspect of animal law is particularly interesting in the context of rights theory. The two dominant rights theories, the will and interest theories, provide differing accounts of who is eligible to be a rights holder.^110^ Pursuant to the will theory, eligibility as a rights holder is dependent upon having control over another person’s duty.^111^ As animals lack this element of agency, conceptually they are not eligible rights holders under this theory.^112^ While this may appear to be a problem for potential animal rights, will theory has been criticised and lacks the basis to constitute an exhaustive explanation of human rights.^113^ One reason for this is that some humans, including infants and significantly cognitively impaired people, likely also lack agency.^114^

Conversely, the interest theory posits that to have rights, an individual must have interests. In this respect, Joel Feinberg asserts that ‘the sorts of beings who *can* have rights are precisely those who have (or can have) interests’.^115^ Further, for the interest to be protected as a right, it must be of sufficient importance to provide a basis for imposing a duty on another being.^116^ As sentient beings can subjectively experience both positive and negative emotions, it can be clearly argued that they have interests. For example, they might have an interest in avoiding pain and suffering. As a result, all sentient beings, including non-human animals, are eligible to be rights holders under this theory.^117^

As is evident from this summary, under the interest theory of rights, the capacity to have interests is frequently linked with sentience. Stucki notes in relation to the interest theory that, ‘Typically, though not invariably, the capacity for having interests in this broad sense is bound up with sentience’.^118^ For example, Kramer argues that rights should attach to sentient beings, because ‘within the scheme of Western values, considerable moral significance attaches to this distinction between living things that are conscious and living things that are nonconscious’.^119^ Equally, where a being lacks sentience and therefore cannot have experiences like joy and happiness or pain and suffering, this is an important factor that suggests that such a being is not eligible to be a rights holder.^120^

### B. Extension of the Interest Theory of Rights to Animals

Following the interest theory of rights, then, animals are potential rights holders. This conclusion has been examined in the literature. For example, Alastair Cochrane draws on Joseph Raz’s conception of interest rights and applies it to animals.^121^ He asserts that ‘Rights possession simply means that their holders have certain important, basic interests that impose duties on others’.^122^ In this respect, Cochrane argues that animals have interests in not suffering and in not being killed. These interests, he argues, are sufficiently important to constitute rights.^123^ Like Cochrane, Tasioulas does not see rationality or agency as a requirement for rights eligibility and acknowledges the potential for animals to be rights holders.^124^

David Favre also makes the connection between animal interests and rights.^125^ He argues that animals have interests that include obtaining adequate food and water, engaging in species-appropriate behaviour, and being free from pain and suffering.^126^ These interests are an integral part of being alive for animals, just as they are for humans. According to Favre, ‘Interests exist as a manifestation of the genes which create the living creatures of this Earth’.^127^ Jurisdictions that have animal welfare laws already recognise these interests. Recognition of such interests have the potential to bring animals ‘into the circle of rights holders’.^128^

### C. Sentience as a Foundation for Interests (and Thus Rights)

It is notable, although not always stated, that, pursuant to this line of argument, interests derive from sentience. As noted above, to be sentient means that an individual experiences the world subjectively and can experience positive and negative states. These experiences or feelings are of importance to the individual; for example, a sentient being will avoid painful experiences and will seek pleasurable ones.^129^ An individual that can experience pain and suffering will have an interest in avoiding pain and suffering; however, an individual that cannot experience those emotions will have no such interest. Similarly, an individual that can experience joy and contentment will have an interest in feeling those emotions, whereas an individual unable to experience positive feelings cannot have an interest in doing so. As interests can be derived from sentience in this way, sentience itself may be used as a *de facto* criterion to determine eligibility as a rights holder under the interest theory of rights.

This connection between interests and sentience is critical to establishing that sentience can provide a foundation for animal rights. Under interest theories of rights, individuals need to have interests to be eligible as rights holders. Some theories, like Tasioulas’s theory discussed above, posit interests themselves as a foundation for human rights. The closeness of the relationship between sentience and interests therefore indicates that if it is correct to talk about interests leading to rights eligibility, it is also correct to talk about sentience giving rise to rights eligibility. If interests can be conceptualised as a foundation for rights, then sentience can provide the normative justification for such a foundation.

## 4. Sentience and the Purpose of Rights

Sentience is also uniquely suited to act as a foundation for legal animal rights because of its direct link to the experience of pain and suffering. It is only by being sentient that an individual can feel pain and suffer. According to some human rights theories, the fundamental purpose of human rights is to prevent pain and suffering.^130^ Similarly, it might be argued that the fundamental purpose of recognising animal rights is to prevent animals’ pain and suffering. This section will explain why it is appropriate to refer to human rights theories in this context and how theories contending that it is the specific interests in avoiding pain and suffering that give rise to rights apply in the context of animals.

### A. Human Rights Theory Is Relevant to Discussing the Purpose of Animal Rights

Various accounts exist as to the foundations of rights, or the ‘grounds or basis or justification’^131^ of rights, which are generally provided in the context of human rights.^132^ Ostensibly, it may seem inappropriate to refer to human rights theories when considering future animal rights. Animals are unlikely to require the same rights as humans.^133^ Nevertheless, several human rights are clearly directed towards the prevention of pain and suffering, for example, the right to life and the right not to be subject to cruel, inhuman or degrading treatment or torture. Moreover, the context within which contemporary human rights discourse arose, being World War II and in particular the Holocaust, was intimately connected with the rejection of the intentional infliction of human pain and suffering.^134^ Like the motivations that underpin some human rights, future animal rights seem to be primarily motivated by the objective of preventing animal pain and suffering. Thus, to the extent that some human rights are directed towards the prevention of pain and suffering and respect for dignity, human rights theory may be instructive when considering the proper foundation for future animal rights.

### B. Prevention of Pain and Suffering Underpins Some Human Rights

The argument that interests give rise to rights may be framed yet more narrowly. In this respect, it can be argued that it is the specific interests in avoiding pain and suffering (including psychological pain and suffering) that give rise to rights.^135^ Philosophers that locate rights in interests in avoiding pain and suffering often draw on the work of Emmanuel Levinas. Costas Douzinas, for example, argues that human rights are grounded in the emotional reaction to preventable suffering.^136^ In this respect, Wheatley’s account of Douzinas’s work is evocative. According to Wheatley, Douzinas argues that

The basis of human rights … is the ethical demands the face of the Other places on me, including the demands of the face of the child whose back is burning from napalm, that of the young man stood in front of a tank, or the face of a women [*sic*] with an emaciated body stood behind the fence of a concentration camp.^137^

Similarly, Upendra Baxi argues that

the historic mission of ‘contemporary’ human rights is *to give voice to human suffering*, to make it visible, and to ameliorate it. The notion that human rights regimes may, or ought to, contribute to the ‘pursuit of happiness’ remains the privilege of a miniscule of humanity. For hundreds of millions of the ‘wretched of the earth,’ human rights enunciations matter, if at all, only if they provide shields against torture and tyranny, deprivation and destitution, pauperization and powerlessness, desexualization and degradation.^138^

As noted above, the conception of rights as being directed towards the avoidance of pain and suffering is supported by the historical context to the enactment of the modern human rights legal system. It was the horrific suffering and brutality endured during the Holocaust and World War II that brought the international community together to draft the Universal Declaration of Human Rights of 1948, which remains the foundational document of international human rights law.^139^

### C. Prevention of Pain and Suffering Would Underpin Future Animal Rights

Similarly, the view that the core concern of rights is the prevention of pain and suffering seems particularly appropriate in the context of fundamental animal rights. Emotive descriptions of pain and suffering experienced by animals farmed in intensive animal agricultural operations and in live export abound. Douzinas’s approach can be easily applied: the face of the other—sheep and cattle packed tightly onto boats and suffering from heat stress, sows confined in sow stalls, chickens living in wire cages smaller than an A4 size piece of paper^140^—places ethical demands on humans because we can recognise and empathise with the pain and suffering experienced by the other. Removing the visibility of these cruelties by limiting the public’s ability to view them^141^ has helped to enable those responsible to avoid preventative action to date. This view of individual rights as profoundly concerned with avoiding unnecessary pain and suffering is just as relevant, perhaps in contemporary society even more so, for animals as it is for humans. The pain and suffering experienced by billions of animals daily means that any fundamental rights for animals should be directed at least in the first instance towards the amelioration of such horror.

This position on the real purpose of rights may be opposed on the basis that it fails to account for the importance of positive states for living beings. It is true that a life without pain and suffering, but also without joy and happiness, is not a life that most humans (or probably animals) seek. In this respect, animal law scholars have praised the recognition of animal sentience on the basis that they see it as importing positive animal welfare obligations on humans.^142^ While traditional animal welfare legislation has focused on the prevention of pain and suffering, sentience recognition has been interpreted as potentially introducing obligations on humans to ensure that animals also enjoy positive states.^143^ Contending that the fundamental purpose of rights is to prevent pain and suffering, however, does not negate the importance of positive states. In some circumstances, the absence of positive obligations may amount to the infliction of suffering. For example, housing hens in battery cages may not constitute a direct infliction of pain or suffering on the hens, but denying the hens the ability to engage in natural behaviours or to socialise with other hens would likely lead the hens to experience psychological suffering.^144^ Moreover, rights are not intended to deliver an optimal life because such a goal is not within the reach of the law. Rather, rights are, at least in part, critical to safeguarding individuals against the actions of others that could lead them to experience very negative states.

## 5. Further Reflections on the Proposed Foundation for Fundamental Animal Rights

### A. The Need for a Two-Level Account and Response to Arguments for Exclusionary Human Dignity

Interests are not generally sufficient on their own to ground rights claims under the interest theory of rights. The rights claimant will also need to matter for their own sake. In other words, the rights claimant must be ‘intrinsically valuable’.^145^ In relation to human beings, the intrinsic value criterion is satisfied by reference to the quality of human dignity. In this respect, human dignity is said to inhere equally in all people.^146^ Following the interest theory of rights criteria, then, all human beings have interests, which makes them eligible as rights holders, and all of them equally have human dignity, meaning that they have inherent value. Thus, under this theory, all human beings are rights holders.

While animals clearly have interests that derive from their sentience, early philosophers argued that they are not entitled to rights because they lack inherent worth. This argument was generally phrased as an interpretation of human dignity that rests on some characteristic of human beings that separates them from animals.^147^ For example, according to Cicero, human rationality gives rise to human dignity,^148^ while according to Kant, human dignity is based on humans’ moral rationality and agency.^149^ The obvious problem with these arguments is that not all humans have these characteristics.^150^ For example, infants or very cognitively disabled people may lack them.^151^ Moreover, it can be argued that some animals do have these characteristics, at least to some degree.^152^ Proponents for denying animal rights have endeavoured to overcome this problem by arguing that the characteristics are typical or within the nature of the human species but no other species.^153^ However, determining what is and is not characteristic of a species is not a clear-cut process and if applied to other non-morally relevant characteristics, such as gender, would lead to outcomes that would not be accepted by most people.^154^

Subsequent theories of human rights argued for foundational concepts like vulnerability, capability, precarity and care.^155^ The potential of such concepts to extend beyond the human species, however, raised the question of whether animals should also have rights.^156^ This is because concepts like these may be applied to both humans and animals; ‘animals are continuous with humans in all of these respects’.^157^ While these arguments constitute a positive development from an animal protection perspective, they may also be perceived as dangerous from a human rights perspective. Human rights discourse often relies on concepts of human dignity and equality to extend rights to all humans, including marginalised and vulnerable groups. If rights are underpinned by facts about human or animal properties, like sentience or vulnerability, then a door is potentially opened to discriminate between humans on this basis. As Will Kymlicka explains, ‘The fear is that if the line between human and animal is blurred, vulnerable human groups will be the ones whose humanity will be put into question’.^158^

Recent times have seen a renewal of arguments for an exclusionary conception of human dignity. This movement, referred to as ‘new dignitarianism’,^159^ seeks to use the idea of human dignity to resist these perceived threats to the unique moral and legal status of humans.^160^ For example, Kateb argues that it is the capacity of humans to break with nature that distinguishes them from other animals.^161^ According to Kateb, human dignity means that ‘All individuals are equal [and that] … no other species is equal to humanity’.^162^ Similarly, Catherine Dupré argues that

The legal system of human rights protection … rests on the assumption that, as human beings, we are born with the unique quality of dignity that distinguishes us from other beings (primarily animals), justifying and explaining the special protection of our rights.^163^

Thus, new dignitarians use the idea of human supremacy over animals to justify attributing rights to all humans, and only humans.

Arguments for exclusionary human dignity, however, suffer from significant problems. First, research indicates that the perceived threats to human rights are not real. As Will Kymlicka explains, emphasising species hierarchy serves to aggravate rather than alleviate dehumanisation.^164^ The more that humans differentiate between humans and animals, the more liable they are to differentiate between humans.^165^ Moreover, the converse is also true: ‘humane education regarding animals—emphasising interspecies affinities and solidarities—is known to encourage greater empathy and pro-social attitudes towards other humans’.^166^ Thus, relying on an exclusionary conception of human dignity in order to protect the human rights of minorities is likely to be counterproductive. Moreover, traditional arguments for exclusionary human dignity based on species-specific characteristics like rationality still suffer from the weaknesses outlined above; there is no characteristic of humans that can be demonstrated to belong to all humans and no animals.

Instead, as Raffael Fasel demonstrates, founding rights on both dignitarian and naturalist grounds is likely to increase the practical viability of a rights theory.^167^ Such an approach is supported by Tasioulas, who states that ‘We should reject as false the choice between a status-based or dignitarian account of human rights and an interest-based account’.^168^ In the context of animal rights, such an approach might involve founding animal rights on interests derived from animal sentience (thus limiting rights holders to sentient animals) and animal dignity and equality. This is the approach advocated in this article.

Arguably, however, these theoretical problems are no longer relevant in terms of the question of fundamental legal animal rights. This is because of the existence of animal welfare laws that seek to protect animals seemingly for their own sakes, indicating that animals are considered to have inherent worth, or ‘intrinsically valuable interests’.^169^ Further, as outlined above, many jurisdictions have gone beyond simply passing animal welfare laws and have included express statements to the effect that animals are inherently valuable. For example, jurisdictions including Switzerland,^170^ Germany^171^ and Austria^172^ have passed laws recognising that animals are not objects. Similarly, jurisdictions including Switzerland and Germany have assigned inherent legal relevance to animals through amendment to federal constitutions.^173^ Other jurisdictions have included statements in legislation recognising that animals have inherent worth.^174^ The Swedish Animal Welfare Act (2018:1192)^175^ states a purpose as promoting respect for animals. Thus, in many of these jurisdictions, it seems that the law has determined that animals do have inherent worth and, pursuant to a theory of rights like Tasioulas’s, this would make them appropriate to be rights holders.

### B. The Need for a Pluralist Approach

This article advocates for a pluralist foundation for future animal rights based on interests derived from sentience and intrinsic worth. The interests concerned are pluralistic in nature. This means that there is no single underpinning interest such as pleasure or liberty. Instead, multiple interests may ground future animal rights. Moreover, as with Tasioulas’s account of the foundation of human rights, ‘The list of interests is open-ended rather than definitively established once-and-for-all’.^176^

Allowing a plurality of interests to ground animal rights is important because it will allow for flexibility of approach. Rather than restricting the focus to a single or overarching interest, any objectively established animal interests could be drawn upon as a basis for a right. As Tasioulas states, ‘a pluralist is free to exploit the rights-generative power of all universal … interests within the discourse of … rights’. This flexibility is critical, considering the incredibly diverse range of sentient animals. Different species are likely to have different interests derived from the nature of their sentience. Thus, providing flexibility to tailor rights to the interests of a species is essential.

Further, allowing an open-ended range of interests to inform animal rights will provide a space for advancements in scientific knowledge to inform rights development. Science in relation to animal sentience has been growing steadily over the past few decades.^177^ Nevertheless, there is still significant research to be done to better understand animal sentience, particularly in terms of positive emotions and in relation to specific species, notably insects.^178^ Allowing a diversity of animal interests to provide a foundation for animal rights enables future developments in scientific knowledge of animal sentience to influence which rights are accorded to animals.

### C. Potential Ambiguity in Terms

The appropriateness of using sentience as a foundation for animal rights may be challenged on the basis that the term itself is too vague. In this respect, Marcelo Rodriguez Ferrere argues that sentience is ‘not self-explanatory, nor is there a clear and incontrovertible definition at present’.^179^ He points to the differences in definitions offered at various points in time and in different jurisdictions as evidence in this respect.^180^ While it is true that the understanding of sentience has changed over time and that the definitions included in different jurisdictions’ legislation vary in significant respects, these are not insurmountable obstacles to utilisation of sentience as a foundation for animal rights. It is inevitable that legislatures will use different terminology for sentience depending on their understanding of what the term means and what they are seeking to achieve. This is only problematic where the definition that is used is so narrow as to prove incapable of supporting claimed rights.^181^ Where the legislature elects not to define the term ‘sentience’, or to leave aspects of the definition open for further interpretation, courts will be responsible for interpreting the word in accordance with the settled principles of statutory interpretation. Where a definition is adopted that fails to reflect a modern understanding of the meaning of sentience, animal advocates should continue to seek law reform to ensure that the recognition of sentience accords with contemporary scientific understandings.

In terms of general non-legislative definitions, Rodriguez Ferrere asserts that today ‘Modern definitions range from complex to far too simplistic’.^182^ Again, although this is true, this article proposes that it is not an impediment to the use of the term as a foundation for animal rights. Some key characteristics of sentience appear to emerge from the various definitions set out in the literature.^183^ These include that sentience involves a capacity to have both positive and negative states, and that these states matter to the individual.^184^ Legislatures should seek to include these elements in their definitions. Courts have extensive experience in interpreting legislation according to the ordinary or technical meaning of the words, interpreted in light of the purposes of the legislation.^185^ They should be trusted to do their job of interpreting sentience provisions in light of the legislative purposes for which they were introduced.

The objection that sentience is too vague a term to ground animal rights echoes the common criticism from human rights literature that ‘Perhaps the phrase “human dignity” is too vague to be of any foundational use’.^186^ For example, Neomi Rao argues that the phrase ‘human dignity’ is used by courts in different ways depending on ‘how they balance individual rights with the demands of social policy and community values’.^187^ Yet, while there are a variety of different definitions of human dignity, essential characteristics underpin these definitions.^188^ Further, the concept has enabled societies to develop an international legal system of human rights protections that can be drawn upon to assist individuals experiencing human rights violations. Similarly, key aspects of sentience emerge from the varying definitions and give the word a core meaning. This essential meaning is important because it provides the dividing line between those things that command moral concern and those things that do not.^189^

## 6. Conclusion

As it stands, the world is an unjust place in which human beings disrespect and exploit other sentient beings and our shared environment. To transition towards a world in which human beings can live in harmony with other animals and the environment with which we are interdependent, deep cultural change is required. Human beings must learn to recognise the intrinsic value of other animals and the environment and develop the law to ensure that such value is respected and protected.

Recent decades have seen trends emerge in mostly Western countries of expressly recognising in the law that animals are sentient and have intrinsic worth. The extent to which such statements are symbolic versus having a real and substantive impact remains unclear.^190^ Nevertheless, even if the legal recognition of animal sentience and intrinsic value in some jurisdictions around the world constitutes mere ‘humane-washing’,^191^ these provisions may yet represent—or be used as—a small step towards the required cultural change. Specifically, animal sentience and intrinsic worth may be drawn upon to provide a foundation for the development of animal rights.

This article contends that the primary purpose of rights is the prevention of needless pain and suffering by sentient beings. As Pocar states, ‘the fact of life itself and its accompanying capacity for suffering provides a sufficient basis for assuming the existence of protectable rights among non-human beings. Here again, we see an evident parallel with human rights’.^192^ When we talk about the foundation of rights, we are referring to the justification or reason for the existence of those rights. In the context of human rights, the concept of human dignity is generally put forward as the justification for rights.

This article asserts that animal sentience and intrinsic worth should be used as a foundation for animal rights. Sentience is not the same thing as intrinsic worth and, on its own, fails to explain why sentient beings are deserving of protection. The concept of intrinsic worth can be used to provide this explanation, in that it asserts that the capacity to subjectively experience the world is precious and deserving of protection. Like the concept of human dignity, which plays a foundational role in human rights law, the concepts of animal sentience and intrinsic worth have the benefit of bringing people to a point of consensus. The proliferation of provisions for the recognition of animal sentience and intrinsic worth in various jurisdictions are indicative of this broad consensus. Further, both sentience and intrinsic worth, like dignity, are not based in any comprehensive religious or moral view, thus increasing the possibility that the terms could be the subject of international consensus.

It might be countered that all animals are not the same in their capacity for pain and suffering or in their intrinsic worth. It is not the position of this article that they are. Instead, the foundation of rights argued for here identifies the minimum characteristics to ground fundamental rights. What rights they ground will depend to some degree on the individual animal they are designed to protect.

## Footnotes

1 John Tasioulas, ‘On the Foundations of Human Rights’ in Rowan Cruft, S Matthew Liao and Massimo Renzo (eds), *Philosophical Foundations of Human Rights* (OUP 2015) 45.

2 Cruel Treatment of Cattle Act 1822.

3 Saskia Stucki and Visa Kurki, ‘Animal Rights’, *Encyclopedia of the Philosophy of Law and Social Philosophy* (2020) 1; Erwin Lengauer, ‘Tom Regan’s Philosophy of Animal Rights: Subjects-of-a-Life in the Context of Discussions of Intrinsic and Inherent Worth’ (2020) 97 Problemos 87, 87.

4 Gonzalo Villanueva, ‘“The Bible” of the Animal Liberation Movement: Peter Singer and Animal Liberation, 1970–1976’ (2016) 13 History Australia 399, 410–11.

5 Donald M Broom, *Sentience and Animal Welfare* (CABI, 2014) 20–1; David J Mellor, ‘Welfare-Aligned Sentience: Enhanced Capacities to Experience, Interact, Anticipate, Choose and Survive’ (2019) 9 Animals 440.

6 eg in Australia, lambs can have folds of skin from their tail area removed without anaesthetic (Animal Health Australia, *Australian Animal Welfare Standards and Guidelines for Sheep* (1st edn, 2016); in the UK, animals can be slaughtered without being stunned where it is for religious purposes (The Welfare of Animals at the Time of Killing (England) Regulations 2015).

7 eg Saskia Stucki, ‘Towards a Theory of Legal Animal Rights: Simple and Fundamental Rights’ (2020) 40 OJLS 533, 533; Martha C Nussbaum, *Frontiers of Justice: Disability Nationality, Species Membership* (Harvard UP 2006) 351–2, 392–401; Gary L Francione, *Introduction to Animal Rights: Your Child or the Dog?* (Temple UP 2000) 151–66; Alasdair Cochrane, *Animal Rights without Liberation: Applied Ethics and Human Obligations* (Columbia UP 2012).

8 Ian Weldon, ‘Why Doesn’t Animal Protection Legislation Protect Animals? (And How It’s Getting Worse)’ (2008) 1 Australian Animal Protection Law Journal 9, 9.

9 Peter Sankoff, ‘The Protection Paradigm: Making the World a Better Place for Animals?’ in Peter Sankoff, Steven White and Celeste Black (eds), *Animal Law in Australasia: Continuing the Dialogue* (2nd edn, The Federation Press 2013) 28–30.

10 Rochelle Morton, Michelle L Hebart and Alexandra L Whittaker, ‘Increasing Maximum Penalties for Animal Welfare Offences in South Australia—Has It Caused Penal Change?’ (2018) 8 Animals 236, 236. For a critique of this approach to animal protection, see Justin Marceau, *Beyond Cages: Animal Law and Criminal Punishment* (CUP 2019).

11 Grundgesetz für die Bundesrepublik Deutschland (Basic Law of the Federal Republic of Germany) (1949), art 20a. In relation to constitutional provisions recognising animals, see Jessica Eisen and Kristen Stilt, ‘Protection and Status of Animals’, *Max Planck Encyclopedia of Comparative Constitutional Law* (OUP 2016).

12 See eg Germany: § 90a BGB Bürgerliches Gesetzbuch since 1990; Austria: Art 285a ABGB Allgemeines bürgerliches Gesetzbuch since 1988; Switzerland: Art 641a ZGB Zivilgesetzbuch since 2002; Czech Republic: Art 494 OZ Občanský Zákoník since 2012; France: Art 515-14 CC Code Civil since 2015; the Netherlands: Art 3:2a BW Burgerlijk Wetboek since 2016; Belgium: Arts 3.38–9 BW Burgerlijk Wetboek since 2020, in force 1 September 2021. In relation to these developments, see Eva Bernet Kempers, ‘Neither Persons nor Things: The Changing Status of Animals in Private Law’ (2021) 1 European Review of Private Law 39.

13 Protocol annexed to the Treaty of the European Community—Protocol on Protection and Welfare of Animals [1997] OJ C340/110.

14 Animal Welfare Act 2008 (TZ), ss 3, 4(b)(i).

15 Animal Welfare Act 1999 (NZ), long title.

16 Animal Welfare and Safety Act, CQLR 2016, c B-3.1, preamble; ‘Quebec Defines Animals as “Sentient Beings” in New Legislation’ (*CTV News*, 4 December 2015) <www.ctvnews.ca/politics/quebec-defines-animals-as-sentient-beings-in-new-legislation-1.2687500> accessed 29 July 2022.

17 OR Rev Stat §167.305 (2021). See generally Jessica Park, ‘Yes, Chickens Have Feelings Too. The Recognition of Animal Sentience Will Address Outdated Animal Protection Laws for Chickens and Other Poultry in the United States’ (2021) 22 San Diego International Law Journal 335.

18 Animal Welfare Act 1992 (ACT), s 4A(1)(a).

19 Ley No 1774 de 2016 (CO), art 1, 665.

20 Tierschutzgesetz 2005 (Animal Welfare Act) (CH), art 3a defines animal dignity as ‘the inherent worth of the animal that must be respected when dealing with it’; Wet dieren 2011 (Animals Act) (NL), art 1.3; Animal Welfare Act 1992 (ACT), s 4A(1)(b); Bundesverfassung 1999 (Federal Constitution) (CH), art 120(2); Tierschutzgesetz 2005 (Animal Welfare Act) (CH), arts 1 and 3(a); Tierschutzgesetz 2010 (Animal Welfare Act) (LI), art 1; Loi sur la protection des animaux 2018 (Animal Welfare Act) (LU), art 1; Wet op de dierproeven 1977 (Experiments on Animals Act) (NL), art 1a; European Parliament and Council Directive 2010/63/EU of 22 September 2010 on the protection of animals used for scientific purposes [2010] OJ L276/33, Recital 12.

21 Stucki (n 7) 543.

22 Tercer Juzgado de Garantı´as de Mendoza 3 November 2016, Expte Nro P-72.254/15.

23 Corte Suprema de Justicia 26 July 2017, AHC4806-2017. However, this ruling was reversed by the Constitutional Court of Columbia: Corte Suprema de Justicia 16 August 2017, STL12651-2017.

24 Supreme Court of India 7 May 2014, civil appeal no 5387 of 2014 [62].

25 Islamabad High Court 21 May 2020, WP no 1155/2019.

26 See Tom Sparks, Visa Kurki and Saskia Stucki, ‘Animal Rights: Interconnections with Human Rights and the Environment’ (2020) 11 JHRE 149, 149–50.

27 ibid 149.

28 Stucki (n 7) 533.

29 ibid 535.

30 ibid 544. See also Visa Kurki, ‘Legal Personhood and Animal Rights’ (2021) 11 Journal of Animal Ethics 47, 50.

31 Stucki (n 7) 552.

32 Cass R Sunstein, ‘Standing for Animals (with Notes on Animal Rights)’ (2000) 47 UCLA Law Review 1333, 1336–7; Clare McCausland, ‘The Five Freedoms of Animal Welfare Are Rights’ (2014) 27 Journal of Agriculture and Environmental Ethics 649; Matthew H Kramer, ‘Do Animals and Dead People Have Legal Rights’ (2001) 14 CJLJ 29.

33 Note that reference to ‘animal rights’ from this point onwards is to fundamental animal rights, unless noted otherwise. As Stucki outlines, while the rights developed in countries such as India include ‘fundamental’ rights such as a right to freedom from torture, they suffer from high infringeability, and as such fall outside the concept of rights considered in this article.

34 In this article, ‘intrinsic worth’ is synonymous with ‘intrinsic value’, ‘inherent value’, ‘inherent worth’ and ‘dignity’.

35 eg an interest in avoiding pain and suffering.

36 See eg Costas Douzinas, The End of Human Rights: Critical Legal Thought at the Turn of the Century (Hart Publishing 2000) 351.

37 Stucki (n 7) 535.

38 Donald M Broom, ‘Animal Welfare and Legislation’ in Frans JM Smulders and Bo Algers (eds), *Welfare of Production Animals: Assessment and Management of Risks* (Wageningen Academic Publishers 2009) 339, 340; David DeGrazia, *Taking Animals Seriously: Mental Life and Moral Status* (CUP 1996) 75; James K Kirkwood, ‘The Distribution of the Capacity for Sentience in the Animal Kingdom’ in Jacky Turner and Joyce D’Silva (eds), *Animals, Ethics and Trade: The Challenge of Animal Sentience* (Routledge 2006) 12; Helen S Proctor, Gemma Carder and Amelia R Cornish, ‘Searching for Animal Sentience: A Systematic Review of the Scientific Literature’ (2013) 3 Animals 882, 882–4; Mellor (n 5) 449–50.

39 Jonathan Birch and others, ‘Review of the Evidence of Sentience in Cephalopod Molluscs and Decapod Crustaceans’ (London School of Economics and Political Science 2021) 17.

40 Broom, Sentience and Animal Welfare (n 5) xiii–xiv.

41 Robert C Jones, ‘Science, Sentience, and Animal Welfare’ (2013) 28 Biology and Philosophy 1, 6.

42 ibid 8; Donald M Broom, ‘Considering Animals’ Feelings’ (2016) 5 Animal Sentience 1, 8.

43 Proctor, Carder and Cornish (n 38) 882–4; Marcelo Rodriguez Ferrere, ‘The (Symbolic) Legislative Recognition of Animal Sentience’ (2022) 28 Animal Law 117, 123. The potential argument that uncertainties around the meaning of sentience may make it inappropriate as a foundation for animal rights is addressed in s 5C below.

44 Mellor (n 5) 449–50.

45 Michael J Zimmerman and Ben Bradley, ‘Intrinsic vs Extrinsic Value’, *The Stanford Encyclopedia of Philosophy* (Spring edn, 2019) <https://plato.stanford.edu/entries/value-intrinsic-extrinsic/#:~:text=The%20intrinsic%20value%20of%20something,a%20variety%20of%20moral%20judgments.> accessed 14 December 2022.

46 See eg Tore Fougner, ‘Engaging the “Animal Question” in International Relations’ (2021) 23 International Studies Review 862, 877; Stucki (n 7) 543.

47 Will Kymlicka, ‘Human Rights without Human Supremacism’ (2018) 48 Canadian Journal of Philosophy 763, 771.

48 David Bilchitz, ‘Moving beyond Arbitrariness: The Legal Personhood and Dignity of Non-human Animals’ (2009) 25 SAJHR 38, 38.

49 Act against Plowing by the Tayle, and Pulling the Wooll off Living Sheep 1635 (IE).

50 Emily Stewart Leavitt and Diane Halverson, ‘The Evolution of Anti-cruelty Laws in the United States’ in *Animals and Their Legal Rights: A Survey of American Laws from 1641 to 1990* (4th edn, Animal Welfare Institute 1990).

51 Cruel Treatment of Cattle Act 1822 and Cruelty to Animals Act 1849 (GB).

52 Sophie Riley, *The Commodification of Farm Animals* (Springer 2022) 63.

53 Jane Kotzmann, ‘Recognising the Sentience of Animals in Law: A Justification and Framework for Australian States and Territories’ (2020) 42 Syd LR 281, 288.

54 For further discussion, see Jane Kotzmann and Morgan Stonebridge, ‘There Is Value in Stating the Obvious: Why United States Legislatures Should Explicitly Recognize Animal Sentience in Their Laws’ (2021) 30 Cornell JL & Pub Pol’y 425, 442–3.

55 Lora Dunn and David B Rosengard, ‘A Dog Is Not a Stereo: The Role of Animal Sentience in Determining the Scope of Owner Privacy Interests under Oregon Law’ (2017) 23 Animal Law 451, 462.

56
 7 USC § 2131 (2018) (emphasis added).

57 Eläinsuojelulaki (Animal Welfare Act) 4/4/247/1996 (FN), § 1.

58 Zakon o Zaščiti Živali (Animal Protection Act) 2007, Uradni at 43/2007 (SI), art 1 (ZZZiv).

59 eg Tierschutzgesetz (Animal Welfare Act) 1972 (DE), §§ 1 and 17; Prevention of Cruelty to Animals Act, No 59 of 1960, India Code; Ustawa o Ochronie Zwierzat (Animal Protection Act) 1997 (PL), art 1(1); ZZZiv, art 3; Hayvanlari Koruma Kanunu (Animal Protection Bill Law) 24 June 2004, No 5199, art 1; Resmî Gazete, 1 July 2004, No 25509 (TR), art 1; Pro Zakhyst Tvaryn Vid Zhorstokoho Povodzhennya (On the Protection of Animals from Cruelty) 2006, No 3447-IV (UA), preamble; Animal Welfare Act 2006 (GB), ch 45, s 1(3)(4); Bundesgesetzblatt für die Republik Österreich (Animal Welfare Act) No 118/2004 (AT), § 5(1); Dyrevelferdsloven (Animal Welfare Act) (2009) No 97 (NO), § 2.

60 Stucki (n 7) 543. See also Thomas G Kelch, ‘A Short History of (Mostly) Western Animal Law: Part II’ (2013) 19 Animal Law Review 347, 348; Bilchitz (n 48) 44.

61 429 P 3d 1053 (2018).

62 ibid 89.

63 ibid 89.

64 [2016] CCT 1/16.

65 ibid 57.

66 For further discussion, see Kotzmann and Stonebridge (n 54) 444–6.

67 Protocol on Protection and Welfare of Animals (n 13).

68 Consolidated Version on the Treaty on the Functioning of the European Union [2012] OJ C326/01, art 13.

69 Animal Welfare Act 2008 (TZ), ss 3 and 4(b)(i).

70 OR Rev Stat §167.305 (2021). See generally Park (n 17).

71 Animal Welfare and Safety Act, CQLR 2016, c B-3.1, preamble; *CTV News* (n 16). For commentary in relation to this enactment, see Michael Lessard, ‘Can Sentience Recognition Protect Animals? Lessons from Quebec’s Animal Law Reform’ (2021) 27 Animal Law 57.

72 Ley No 1774 de 2016 (CO), art 1, 665; Carlos Contreras, ‘Sentient Beings Protected by Law: Analysis of Recent Changes in Colombian Animal Welfare Legislation’ (2016) 2 Global Journal of Animal Law 1, 3.

73 Wet dieren 2011 (Animals Act) (NL), art 1.3.

74 Gyvūnų Gerovės ir Apsaugos Įstatymas (Law on Welfare and Protection of Animals) No XI-2271, Valstybės Žinios, 20 October 2012 No 122-6216 (LT) (2012), art 1(a).

75 Code Civil (Civil Code) (FR), art 515-14. See generally Jean-Marc Neumann, ‘The Legal Status of Animals in the French Civil Code’ (2015) 1 Global Journal of Animal Law 1.

76 Animal Welfare Act 2018 (SE); Government Bill (SE) No 2017/18, 147.

77 Law Relating to the Protection and Welfare of Animals 2018 (Brussels), art 1.

78 Animal Welfare Act 2020 (DK), art 1.

79 Civil Code of Catalonia (Ley de 2006), art 511-1 (3). In relation to Spain, see Marita Gimenez-Candela, ‘The De-objectification of Animals in the Spanish Civil Code’ (2018) XV Journal of Animal & Natural Resource Law 145. In a similar development, several countries have amended their laws to state that animals are ‘not things’; see Birgitta Wahlberg, ‘Animal Law in General and Animal Rights in Particular’ (2021) 67 Scandinavian Studies in Law 13, 17–18.

80 Animal Welfare Act 1999 (NZ), long title.

81 Animal Welfare Act 1992 (ACT), s 4A(1)(a).

82 Animal Welfare Sentience Act 2022 (GB), ss 1–2.

83 Federal Constitution of the Swiss Confederation (1999), art 120(2); Eva Bernet Kempers, ‘Animal Dignity and the Law: Potential, Problems and Possible Implications’ (2020) 41 Liverpool Law Review 173, 174.

84 Animal Welfare Act (Tierschutzgesetz), art 1.

85 ibid art 3(a).

86 Law Relating to the Protection and Welfare of Animals 2018 (Brussels), art 1; Kempers, ‘Animal Dignity and the Law’ (n 83).

87 Wet dieren 2011 (Animals Act) (NL), art 1.3: ‘(1) De intrinsieke waarde van het dier wordt erkend. (2) Onder erkenning van de intrinsieke waarde als bedoeld in het eerste lid wordt verstaan erkenning van de eigenwaarde van dieren, zijnde wezens met gevoel’ (Google Chrome translation).

88 Animal Welfare Act (Dyrevelferdsloven) (NO), §3. For English version, see <www.regjeringen.no/en/dokumenter/animal-welfare-act/id571188/> accessed 14 December 2022.

89 Animal Welfare Act 1992 (ACT), s 4A(1).

90 High Court of Kerala, 6 June 2000, AIR 2000 KER 340 [13]. See also the partly concurring and partly dissenting opinion of judge Pinto de Albuquerque to the judgment of the European Court of Human Rights in *Herrmann v Germany* App no 9300/07 (ECHR, 26 June 2012); *X und Y gegen Gesundheitsdirektion des Kantons Zürich und Mit* Swiss Federal Supreme Court (7 October 2009) BGE 135 II 384, 405; *Ordonnantie tot wijziging van de wet van 14 augustus 1986 betreffende de bescherming en het welzijn der dieren houdende erkenning van het specifiek statuut voor het dier* (Brussels Hoofdstedelijk Gewest) *BS* 17 December 2018, 99411; *Let the Animals Live v Hamat Gader Spa Village Inc* Israeli Supreme Court (22 June 1997) 1648/96, 45. *Nonhuman Rights Project (Happy) v Breheny* (19 February 2020) Supreme Court of the State of New York, No 260441/2019, 16.

91
*Nonhuman Rights Project (Happy) v Breheny* (n 90) 16.

92 OR Rev Stat §167.305 (2021). See generally Park (n 17).

93 Rodriguez Ferrere (n 43) 125.

94 See eg Animal Welfare Act 1999 (NZ), long title.

95 Acts Interpretation Act 1901 (Cth), s 15AA (Australia); DC Pearce, *Statutory Interpretation in Australia* (9th edn, LexisNexis Butterworths 2019); Brian G Slocum, *Ordinary Meaning: A Theory of the Most Fundamental Principle of Legal Interpretation* (University of Chicago Press 2015).

96 Pearce (n 95) 80.

97 Charlotte E Blattner, ‘The Recognition of Animal Sentience by the Law’ (2019) 9 Journal of Animal Ethics 121, 129–30.

98 Pearce (n 95) 6.

99 eg in relation to New Zealand’s recognition of animal sentience in the Animal Welfare Act 1999 (NZ), long title, Hansard records clearly indicate a concern with the ethical treatment of animals given their sentience, suggesting that sentience implies moral value (New Zealand, Animal Welfare Amendment Bill 2013, House of Representatives, 27 August 2013 (Phil Twyford, John Banks); New Zealand, Animal Welfare Amendment Bill 2013, House of Representatives, 30 April 2015 (Jonathan Young)). Somewhat relatedly, in the US case of *State v Fessenden*, the court rejected an argument that because animals are legal property, they do not have inherent value. Instead, the court found that the horse’s capacity to suffer justified a warrant exception (333 P.3d 282, 284 (Or)). In relation to this case, see Dunn and Rosengard (n 55) 460–1.

100 Mirko Bagaric and James Allan, ‘The Vacuous Concept of Dignity’ (2006) 5 Journal of Human Rights 257, 260.

101 Animal Welfare Act (Tierschutzgesetz), art 3(a).

102
*X und Y gegen Gesundheitsdirektion des Kantons Zürich und Mit* (n 90) 405; Kempers, ‘Animal Dignity and the Law’ (n 83) 184.

103 Animals Act 2011 (Netherlands), art 1.3.

104 Explanatory Memorandum Animals Act 2007–2008, 31 389 (Memorie van Toelichting Wet Dieren) (Netherlands), no 3, 20; Kempers, ‘Animal Dignity and the Law’ (n 83) 187.

105 Kempers, ‘Animal Dignity and the Law’ (n 83) 185.

106 Andrew N Rowan and others, ‘Animal Sentience: History, Science, and Politics’ (2021) 31 Animal Sentience 1, 10.

107 ibid 11; Protocol on Protection and Welfare of Animals (n 13).

108 Jeremy Bentham, Theory of Legislation (R Hildreth tr, CK Ogden ed, Kegan Paul, Trench, Trübner & Co, 1931) ch XVI, 429..

109 Jeremy Bentham, ‘Anarchical Fallacies’ in J Waldron (ed), *Nonsense Upon Stilts: Bentham, Burke and Marx on the Rights of Man* (Methuen 1987) 53.

110 For an overview of these theories, see generally Visa AJ Kurki, ‘Rights, Harming and Wronging: A Restatement of the Interest Theory’ (2018) 38 OJLS 430; Matthew H Kramer and Hillel Steiner, ‘Theories of Rights: Is There a Third Way?’ (2007) 27 OJLS 281; Gary L Francione, *Animals, Property, and the Law* (Temple UP 1995) 95–104.

111 See generally Matthew H Kramer, NE Simmonds and Hillel Steiner, *A Debate over Rights Philosophical Enquiries* (1998 OUP); Kurki, ‘Legal Personhood and Animal Rights’ (n 30) (2021) 51.

112 Stucki (n 7) 540.

113 Visa AJ Kurki, ‘Why Things Can Hold Rights: Reconceptualizing the Legal Person’ in Visa AJ Kurki and T Pietrzykowski (eds), *Legal Personhood: Animals, Artificial Intelligence and the Unborn* (Springer 2017) 82; Kramer and Steiner (n 110) 298; Neil MacCormick, *Legal Right and Social Democracy: Essays in Legal and Political Philosophy* (OUP 1984) 154–66.

114 Joseph Bowen, ‘Beyond Normative Control: Against the Will Theory of Rights’ (2020) 50 Canadian Journal of Philosophy 427, 432.

115 Joel Feinberg, ‘The Rights of Animals and Unborn Generations’ in William T Blackstone (ed), *Philosophy & Environmental Crisis* (University of Georgia Press 1974) 167.

116 Cochrane (n 7) 42. See also Joseph Raz, *The Morality of Freedom* (OUP 1986) 335.

117 Kramer (n 32); Feinberg (n 115).

118 Stucki (n 7) 542–3.

119 Kramer (n 32) 35. See also Leif Wenar, ‘The Nature of Claim-Rights’ (2013) 123 Ethics 202, 227.

120 Kramer (n 32) 36.

121 Cochrane (n 7) 41–2.

122 ibid 2.

123 ibid 83–4.

124 Tasioulas, ‘On the Foundations of Human Rights’ (n 1) 106.

125 David Favre, *The Future of Animal Law* (Edward Elgar Publishing 2021).

126 ibid 50.

127 ibid 50.

128 ibid 50.

129 Blattner (n 97) 125.

130 See the discussion at s 4B below.

131 Tasioulas, ‘On the Foundations of Human Rights’ (n 1) 104–5.

132 See eg ibid 104; John Tasioulas, ‘Human Rights, Legitimacy, and International Law’ (2013) 58 Am J Juris 1, 2; John Tasioulas, ‘The Moral Reality of Human Rights’ in Thomas Pogge (ed), *Freedom from Poverty as a Human Right* (OUP 2007) 77; John Tasioulas, ‘Towards a Philosophy of Human Rights’ (2012) 65 CLP 1, 7; James Griffin, *On Human Rights* (OUP 2008) 152, 250; Alan Gewirth, *Human Rights: Essays on Justification and Applications* (University of Chicago Press 1982) ch 1; Samantha Besson, ‘Justifications’ in Daniel Moeckli, Sangeeta Shah and Sandesh Sivakumaran (eds), *International Human Rights Law* (2nd edn, OUP 2014) 46; Oscar Schachter, ‘Human Dignity as a Normative Concept’ (1983) 77 AJIL 848, 849; Steven Pinker, ‘The Stupidity of Dignity’ (*The New Republic*, 28 May 2008) <https://newrepublic.com/article/64674/the-stupidity-dignity> accessed 31 July 2022; Ruth Macklin, ‘Dignity is a Useless Concept’ (2003) 327 British Medical Journal 1419; S Matthew Liao, ‘Human Rights as Fundamental Conditions for a Good Life’ in Cruft, Liao and Renzo (n 1) 120; James W Nickel, *Making Sense of Human Rights* (2nd edn, Blackwell Publishing 2007) 62; Allen Buchanan, ‘The Egalitarianism of Human Rights’ (2010) 120 Ethics 679, 679–708; Rainer Forst, ‘The Justification of Human Rights and the Basic Right to Justification: A Reflexive Approach’ (2010) 120 Ethics 711, 713; James W Nickel, ‘Personal Deserts and Human Rights’ in Cruft, Liao and Renzo (n 1) 165; Joseph Raz, *The Morality of Freedom* (OUP 1986) 336.

133 Bernard E Rollin, ‘Animal Rights as a Mainstream Phenomenon’ (2011) 1 Animals 102, 113.

134 See eg the preamble of the Universal Declaration of Human Rights (adopted 10 December 1948, UNGA Res 217 A(III)): ‘Whereas disregard and contempt for human rights have resulted in barbarous acts which have outraged the conscience of mankind, and the advent of a world in which human beings shall enjoy freedom of speech and belief and freedom from fear and want has been proclaimed as the highest aspiration of the common people.’

135 See eg Stephen Wheatley, *The Idea of International Human Rights Law* (OUP 2019) 25.

136 Douzinas (n 36) 351.

137 ibid 351, as quoted in Wheatley (n 135) 25.

138 Upendra Baxi, ‘Voices of Suffering and the Future of Human Rights’ (1998) 8 Transnational Law & Contemporary Problems 125, 127.

139 United Nations, ‘History of the Declaration’ <www.un.org/en/about-us/udhr/history-of-the-declaration> accessed 11 October 2022; Jacob Dolinger, ‘The Failure of the Universal Declaration of Human Rights’ (2016) 47 University of Miami Inter-American Law Review 164, 164.

140 This is a very small selection of an extremely long list of abuses inflicted on animals by humans.

141 By eg enacting ‘ag-gag’ laws that seek to prevent animal protection advocates from recording such cruelties or from making recordings public. The Criminal Code Amendment (Agricultural Protection) Act 2019 (Cth) is an example of such a law.

142 See eg Ian A Robertson and Daniel Goldsworthy, ‘Will Victoria Miss the Sentience Doorway and Have a 2021 Animal Law Reform that Is Outdated even before It Is Enacted?’ (2021) <https://papers.ssrn.com/sol3/papers.cfm?abstract_id=3861352> accessed 11 October 2022.

143 ibid 12.

144 Sara Shields and Ian JH Duncan, ‘A Comparison of the Welfare of Hens in Battery Cages and Alternative Systems’ (WellBeing International 2009) 18.

145 Stucki (n 7) 542.

146 Jane Kotzmann and Cassandra Seery, ‘Dignity in International Human Rights Law: Potential Applicability in Relation to International Recognition of Animal Rights’ (2017) 26 Michigan State International Law Review 1, 14.

147 See Sonia Waisman, Pamela Frasch and Bruce Wagman, *Animal Law: Cases and Materials* (5th edn, Carolina Academic Press 2014) 48.

148 Thomas Finegan, ‘Conceptual Foundations of the Universal Declaration of Human Rights: Human Rights, Human Dignity and Personhood’ (2012) 37 Australian Journal of Legal Philosophy 182, 202; Michael Rosen, *Dignity: Its History and Meaning* (Harvard UP 2012) 11–12.

149 Philipp Balzer and others, ‘Two Concepts of Dignity for Humans and Non-human Organisms in the Context of Genetic Engineering’ (2000) 13 Journal of Agricultural and Environmental Ethics 7, 9; Nussbaum (n 7) 131; James Rachels, *The Elements of Moral Philosophy* (1st edn, Temple UP 1986) 114; Rosen (n 148) 30.

150 Valerio Pocar, ‘Animal Rights: A Socio-legal Perspective’ (1992) 19 Journal of Law and Sociology 214, 220.

151 Mirko Bagaric and Keith Akers, *Humanising Animals: Civilising People* (CCH Australia 2012) 32.

152 Paola Cavalieri, The Animal Question: Why Nonhuman Animals Deserve Rights (Catherine Woollard tr, OUP 2002) 78.

153 For further discussion, see Kotzmann and Seery (n 146) 33–5.

154 Peter Singer, *Animal Liberation* (2nd edn, Random House 1990) 240–1.

155 Kymlicka (n 47) 767.

156 ibid 766.

157 ibid 8–9.

158 ibid 773.

159 ibid 768; Raffael N Fasel, ‘The Old “New” Dignitarianism’ (2019) 25 Res Publica 531, 532; Timothy Caulfield and Roger Brownsword, ‘Human Dignity: A Guide to Policy Making in the Biotechnology Era?’ (2006) 7 Nature Reviews 72, 72.

160 Fasel (n 159) 532.

161 George Kateb, *Human Rights and Dignity* (Harvard UP 2011) x.

162 ibid 6.

163 Catherine Dupré, T*he Age of Dignity: Human Rights and Constitutionalism in Europe* (Hart Publishing 2015) 28.

164 Kymlicka (n 47) 772–4. See also Gordon Hodson, Cara MacInnis and Kimberly Costello, ‘(Over)Valuing “Humanness” as an Aggravator of Intergroup Prejudices and Discrimination’ in Paul Bain, Jeroen Vaes and Jacques Philippe Leyens (eds), *Humanness and Dehumanization* (Psychology Press 2014) 106.

165 Kymlicka (n 47) 773.

166 ibid 774.

167 Fasel (n 159) 550.

168 Tasioulas, ‘On the Foundations of Human Rights’ (n 1) 56.

169 Stucki (n 7) 543.

170 Schweizerisches Zivilgesetzbuch (Swiss Civil Code), art 641a.

171 Bürgerlichen Gesetzbuches (German Civil Code), § 90a.

172 Allgemeines bürgerliches Gesetzbuch (Austrian Civil Code), art 285.

173 Gieri Bollinger, ‘Legal Protection of Animal Dignity in Switzerland: Status Quo and Future Perspectives’ (2016) 22 Animal Law 311, 313; Kempers, ‘Animal Dignity and the Law’ (n 83) 185.

174 Animal Welfare Act 1992 (ACT); Wet dieren 2011 (Animals Act) (NL), art 1.3.

175 s 1.

176 Tasioulas, ‘On the Foundations of Human Rights’ (n 1) 51 (emphasis removed). Martha Nussbaum’s capabilities approach for animals bears some similarities to this approach: Martha C Nussbaum, ‘Beyond “Compassion and Humanity”: Justice for Nonhuman Animals’ in Cass R Sunstein and Martha C Nussbaum (eds), *Animal Rights: Current Debates and New Directions* (OUP 2004) 299–319.

177 Helen Proctor, ‘Animal Sentience: Where Are We and Where Are We Heading?’ (2012) 2 Animals 628, 635.

178 Heather Browning and Jonathan Birch, ‘Animal Sentience’ (2022) 17 Philosophy Compass 1.

179 Rodriguez Ferrere (n 43) 124.

180 eg ‘Until around fifteen years ago, sentience was “conceptualized mainly in terms of animals’ capacity to have negative affective experiences, especially pain and any associated suffering,” whereas the capacity of animals to feel positive experiences is now considered possible and important’: Rodriguez Ferrere (n 43) 123.

181 eg where the legislation is expressed to apply exclusively to owned animals, thus excluding wild animals (although sentient), this may mean subsequent recognition of animal rights based on sentience lacks coherency. See art 9 of Loi n° 76-629 du 10 juillet 1976 relative à la protection de la nature (Law 76-629 of 1976 on the Protection of Nature) (FR).

182 Rodriguez Ferrere (n 43) 123.

183 In contrast with those definitions that appear in legislation.

184 Blattner (n 97) 125. In terms of definitions, see eg Mellor (n 5) 449–50, who provides the following definition: ‘a capacity to consciously perceive negative or positive sensations, feelings, emotions or other subjective experiences which matter to the animal’. This aligns with the definition proposed by Ian Robertson and Daniel Goldsworthy as appropriate for legislation, being ‘Sentience means that animals experience negative and positive [physical, mental and emotional] states’. Robertson and Goldsworthy further contend that best practice legislation would also define ‘negative states’ and ‘positive states’, and would substitute any legislative references to ‘pain, distress and suffering’ with the phrase ‘negative and positive states’: Robertson and Goldsworthy (n 142).

185 eg s 15AA of the Acts Interpretation Act 1901 (Cth) (Australia) provides that ‘In interpreting a provision of an Act, the interpretation that would best achieve the purpose or object of the Act (whether or not that purpose or object is expressly stated in the Act) is to be preferred to each other interpretation’. See Kotzmann (n 53) for discussion of the potential of sentience recognition provisions to impact statutory interpretation of animal welfare legislation.

186 Jeremy Waldron, ‘Is Dignity the Foundation of Human Rights?’ in Cruft, Liao and Renzo (n 1) 168.

187 Neomi Rao, ‘Three Concepts of Dignity in Constitutional Law’ (2011) 86 Notre Dame L Rev 183; Neomi Rao, ‘On the Use and Abuse of Dignity in Constitutional Law’ (2008) 14 Columbia Journal of European Law 201, 208.

188 Kotzmann and Seery (n 146) 30–3.

189 This does not mean that non-sentient things lack intrinsic worth and should not be respected. However, it does mean that each *individual* sentient being requires respect, because they experience the world subjectively.

190 Rodriguez Ferrere (n 43).

191 Marc Bekoff, ‘Time to Stop Pretending We Don’t Know Other Animals Are Sentient Beings’ (2022) 31 Animal Sentience 2.

192 Pocar (n 150) 223. See also Baxi (n 138) 146.

## Notes

Many thanks to Morgan Stonebridge for her outstanding research assistance that contributed to this article, and to Professor Dan Meagher for his extremely helpful feedback on previous drafts. All errors remain mine alone.

